# DeepCLEM: automated registration for correlative light and electron microscopy using deep learning

**DOI:** 10.12688/f1000research.27158.2

**Published:** 2022-08-02

**Authors:** Rick Seifert, Sebastian M. Markert, Sebastian Britz, Veronika Perschin, Christoph Erbacher, Christian Stigloher, Philip Kollmannsberger

**Affiliations:** 1 Center for Computational and Theoretical Biology, University of Würzburg, Würzburg, 97074, Germany; 2 Imaging Core Facility, Biocenter, University of Würzburg, Würzburg, 97074, Germany; 3 Department of Neurology, University of Würzburg, Würzburg, 97074, Germany

**Keywords:** Correlative Microscopy, Image Registration, In-silico labeling, Deep Learning

## Abstract

In correlative light and electron microscopy (CLEM), the fluorescent images must be registered to the EM images with high precision. Due to the different contrast of EM and fluorescence images, automated correlation-based alignment is not directly possible, and registration is often done by hand using a fluorescent stain, or semi-automatically with fiducial markers. We introduce “DeepCLEM”, a fully automated CLEM registration workflow. A convolutional neural network predicts the fluorescent signal from the EM images, which is then automatically registered to the experimentally measured chromatin signal from the sample using correlation-based alignment. The complete workflow is available as a Fiji plugin and could in principle be adapted for other imaging modalities as well as for 3D stacks.

## Introduction

Correlative Light and Electron Microscopy (CLEM) combines the high resolution of electron microscopy (EM) with the molecular specificity of fluorescence microscopy. In super-resolution array tomography (srAT) for example, serial sections are imaged first under the fluorescence microscope using super-resolution techniques such as structured illumination microscopy (SIM), and then in the electron microscope ^1^. With this technique, it is possible to identify and assign molecular identities to subcellular structures such as electrical synapses ^1, 2^ or microdomains in bacterial membranes ^3^ that cannot be resolved by EM due to insufficient contrast.

To visualize and interpret the results of CLEM, the fluorescent images must be registered to the EM images with high accuracy and precision. Due to the different contrasts of EM and fluorescence images, automated correlation-based image alignment, as used e.g. for aligning EM serial sections ^4^, is not directly possible. Registration is often done by hand using a fluorescent chromatin stain ^2^, or semi-automatically with fiducial markers using tools such as eC-CLEM ^5^. Further improvement and automation of the registration process is of great interest to make CLEM scalable to larger datasets.

Deep Learning using convolutional neural networks (CNNs) has become a powerful tool for various tasks in microscopy, including denoising and deconvolution as well as classification and segmentation, reviewed in 6 and 7. One interesting application of CNNs is the prediction of fluorescent labels from transmitted light images of cells, also called “in silico labeling” ^8, 9^.

We show here that this approach can be used to predict the fluorescent chromatin stain in electron microscopy images of cell nuclei. The predicted “ *in silico*” chromatin images are sufficiently similar to real experimental chromatin images acquired with SIM to use them for automated correlation-based registration of CLEM images. Based on this observation, we developed “DeepCLEM”, a fully automated CLEM registration workflow implemented in Fiji ^10^ and based on CNNs.

## Methods

### Data acquisition

We used previously acquired imaging data of *Caenorhabditis elegans* and of human skin samples from healthy subjects. Sample preparation as well as the acquisition of the imaging data has been previously described in detail ^1, 2, 11^. Briefly, *C. elegans* worms were cryo-immobilized via high-pressure freezing and subsequently processed by freeze substitution. All samples were embedded in methacrylate resin and sectioned at 100 nm. Ribbons of consecutive sections were attached to glass slides and labeled with fluorophores. Live Hoechst 33342 was used to stain chromatin and immunolabeling was used to visualize molecular identities. The sections were then imaged with SIM super-resolution microscopy. Next, they were processed for electron microscopy by heavy metal contrasting and carbon coating. The regions of interest previously imaged with SIM were then imaged again on the same sections with scanning electron microscopy, resulting in pairs of images that needed to be correlated.

### Manual registration

To prepare ground truth for network training, we manually registered the chromatin channel to the EM images as described in 2. We selected 30 subimages and super-imposed them in the software Inkscape. By reducing the opacity of the chromatin images, they could be manually resized, rotated and dragged until the Hoechst signal coincided with the electron-dense heterochromatin puncta in the underlying EM images. To generate own training data, reproducible methods retaining a record of all transforms are recommended.

### Implementation

**Figure 1.**
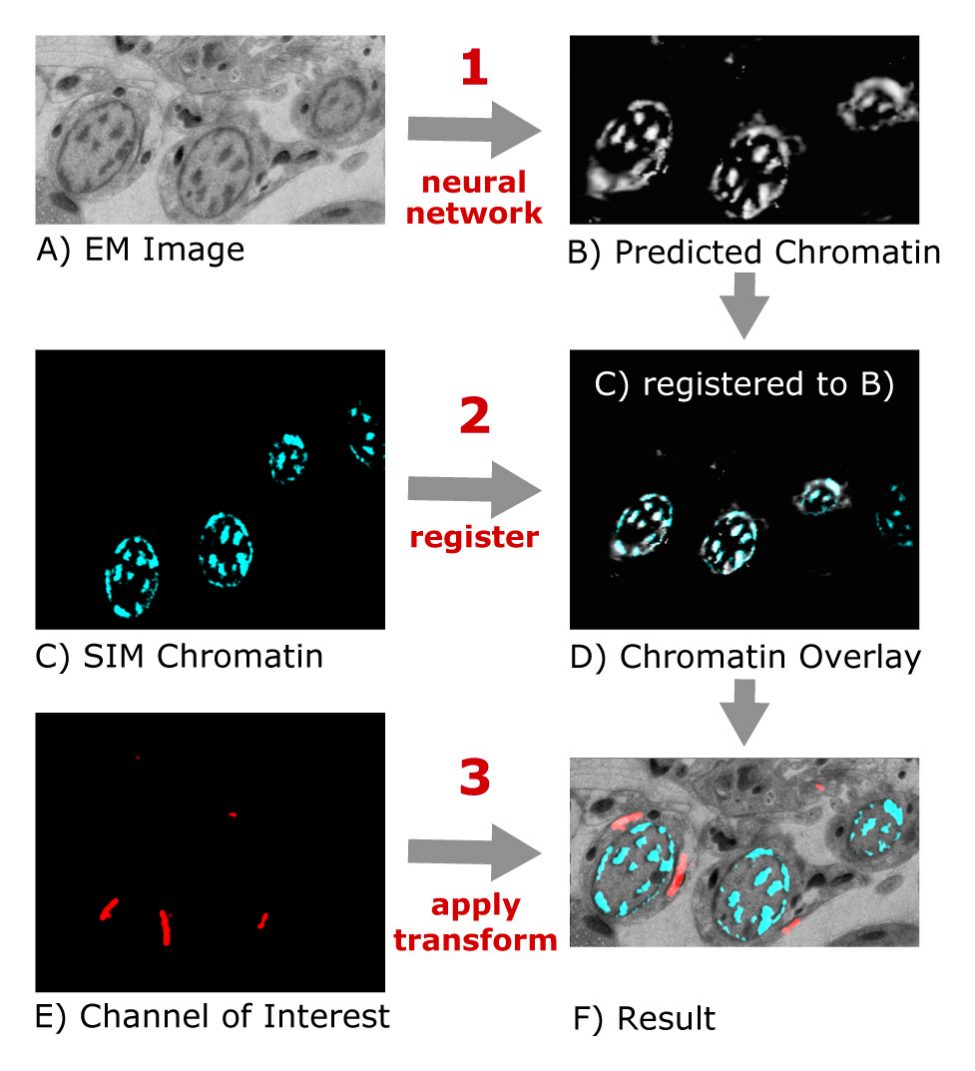
Schematic of the "DeepCLEM" workflow. From the EM image ( **A**), a CNN predicts the chromatin channel ( **B**), to which the SIM image ( **C**) is registered ( **D**). The same transform is applied to the channel of interest ( **E**) to obtain a CLEM overlay ( **F**).

We implemented DeepCLEM as a Fiji ^10^ plugin, using CSBDeep ^12^ for network prediction. Preprocessing of the images as well as network training were performed in Python using scikit-image ^13^ and TensorFlow ^14^. First, a neural network trained on manually registered image pairs predicts the fluorescent chromatin signal from previously unseen EM images ( Figure 1A). This "virtual" fluorescent chromatin image is then automatically registered to the experimentally measured chromatin signal from the sample using the “similarity” transform of the “Register Virtual Stack Slices” plugin in Fiji ( Figure 1B). The transformation parameters from this automated alignment are finally used to register the other SIM images that contain the signals of interest to the EM image ( Figure 1C).

### Operation

**Figure 2.**
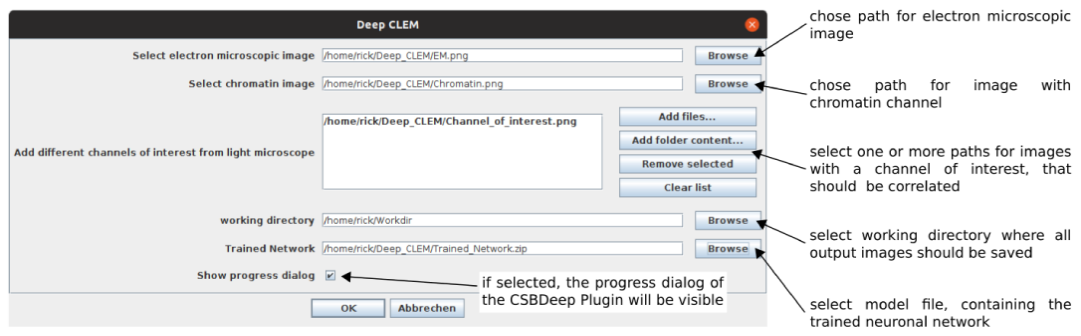
GUI and input parameters for "DeepCLEM".

DeepCLEM requires Fiji ^10^ with CSBDeep ^12^ to run. The paths to the images and model file are entered in a user dialog ( Figure 2). After running DeepCLEM, the correlated images and a .XML file containing the transform parameters are written to the output directory. The workflow is summarized in Figure 1; instructions for installing and running DeepCLEM are included in the repository. The network included in DeepCLEM was trained on in-house data and may work on images with similar contrast, but in most cases, re-training will be necessary – details on the workflow and training parameters are given in a Jupyter notebook in the repository. Running this notebook on a directory with 30-40 aligned ground truth image pairs will yield a model file that can be loaded in the DeepCLEM Fiji plugin.

## Results

### Comparison of network architectures

We trained DeepCLEM on correlative EM and SIM images of *C. elegans* and on human skin tissue and compared prediction and registration results for different network architectures and preprocessing routines. A generative adversarial network (pix2pix) showed promising results in some images from the skin dataset, but overall performance was best using the ProjectionCARE network from CSBDeep ^12^.

### Optimization of preprocessing

EM images had large differences in contrast even when acquired in the same laboratory. We compared different preprocessing routines, including normalization and histogram equalization, and found that standard histogram equalization in Fiji resulted in the best performance on our data. The best combination of preprocessing steps for optimizing contrast may however depend on the data. 

### Quantitative evaluation

We performed a quantification of the quality of the registration on four manually aligned images from an independent experiment, and applying a known shift or rotation. In 75% of cases the registration worked and had a very small error, while in 25% it was completely off by several 100 nm ( Table 1). If two of the test images were included in the training set, the error was much lower, so DeepCLEM works best if a small number of images of each experiment are manually aligned and added to the training data. The remaining images are then reliably aligned. We also varied the number of images in the training set and found that 30-40 ground truth images are sufficient to obtain good alignment on the test set.

**Table 1.** Quantitative evaluation. When applying DeepCLEM on images from a different experiment not represented in the training data, registration failed in 25% of cases (top part, image 1–4). If two manually aligned images were included in the training set, all other test images were successfully registered (bottom part, images 1–2).

Absolute error [nm]								
	aligned		shift in X (3125 nm)		rotation 90°		rotation 180°	
	X	Y	X	Y	X	Y	X	Y
No images from same experiment included in training:								
image 1	65,2	54,0	287,9	215,7	12,2	2,0	102,9	84,8
image 2	69,9	31,0	9234,7	3440,5	276,8	72,0	271,4	1601,1
image 3	713,1	288,0	136,3	65,9	321,3	3,2	77,3	47,5
image 4	103,6	86,8	160,2	158,0	5771,9	4168,6	8861,2	1776,9
Two images from same experiment included in training:								
image 1	33,5	181,0	61,9	100,1	101,7	113,8	115,9	155,1
image 2	16,3	48,9	27,7	20,2	59,9	20,2	84,2	63,9

## Discussion

We developed “DeepCLEM”, a fully automated CLEM registration workflow implemented in Fiji ^10^ based on prediction of the chromatin stain from EM images using CNNs. Our registration workflow can easily be included in existing CLEM routines or adapted for imaging methods other than srAT where corresponding 2D slices need to be registered. If direct prediction of one modality from the other does not work, an alternative is to predict a common representation of both modalities, as described in Ref 15. While we found that "DeepCLEM" performs well under various conditions, it has some limitations: using chromatin staining for correlation requires the presence of at least three heterochromatin patches in the field of view. This limitation could be overcome by using e.g. propidium iodide to label the overall structure of the tissue. Widefield microscopy could be used where SIM is not available, but alignment quality is bounded by the lower-resolution channel.

The popular CLEM registration tool eC-CLEM ^5^ has an “autofinder” function that detects corresponding features using spot finding or segmented regions. We did not perform a direct comparison, but results should be similar if suitable spots are found. If not, then image-to-image translation with DeepCLEM followed by point-based registration in eC-CLEM could be a promising alternative.

## Data availability

Source code, pretrained networks and example data as well as documentation are available online at:

https://github.com/CIA-CCTB/Deep_CLEM.

## Software availability

**Source code available from:** https://github.com/CIA-CCTB/Deep_CLEM.

**Archived source code at time of publication:** https://doi.org/10.5281/zenodo.4095247 ^16^

**License:** MIT License.
