# Supplementary material for: DeepCLEM: automated registration for correlative light and electron microscopy using deep learning
Source: F1000Res. 2023 Dec 28;9:1275. Originally published 2020 Oct 27. [Version 3] doi: 10.12688/f1000research.27158.3 (PMC10311120; doi:10.12688/f1000research.27158.3)
Supplement: Peer review history

# Reviewer report: Reviewer response for version 3

**DOI:** 10.5256/f1000research.160461.r234014

**Published:** 2024-01-25

**Authors:** Martin L. Jones

**Affiliations:** 1 Electron Microscopy Science Technology Platform, The Francis Crick Institute, London, UK

The addition of more novice-focused documentation is a good addition.

Are the conclusions about the tool and its performance adequately supported by the findings presented in the article?

Partly

Is the rationale for developing the new software tool clearly explained?

Yes

Is the description of the software tool technically sound?

Partly

Are sufficient details of the code, methods and analysis (if applicable) provided to allow replication of the software development and its use by others?

Partly

Is sufficient information provided to allow interpretation of the expected output datasets and any results generated using the tool?

Yes

Reviewer Expertise:

Image Analysis, CLEM, Machine Learning, Electron microscopy

I confirm that I have read this submission and believe that I have an appropriate level of expertise to confirm that it is of an acceptable scientific standard.

# Reviewer report: Reviewer response for version 2

**DOI:** 10.5256/f1000research.136822.r179427

**Published:** 2023-06-29

**Authors:** Christopher Schmied

**Affiliations:** 1 AG Han Sun, Leibniz-Forschungsinstitut für Molekulare Pharmakologie, Berlin, Berlin, Germany

The authors present an image registration workflow to register light microscopy (LM) images into electron microscopy (EM) images for correlative light-electron microscopy (CLEM). To solve the multimodal registration problem, DeepCLEM predicts a LM image from the EM image using ProjectionCARE. The predicted LM image is then used to compute the necessary transformation of the LM image to the EM image. Apart from the pre-trained model, which is limited to data it has been trained on, the authors also describe how to train the model on their own data. 

 Overall, a very nice approach that has been well implemented.

 In my review I verified if the provided software package and its documentation allows the successful reproduction of the described workflow. I also assessed if the provided article sufficiently describes and discusses the proposed approach (i.e. scientific context and limitations). 

**Application of the pre-trained model:**

 I was able to easily install the software as per documentation. The provided test images allow to easily verify the correct operation of the Fiji workflow. The output data is clearly and sufficiently described. Positive is that the transformation file is provided that allows to apply the transformation on other data.

 I found some points problematic in the documentation:

 "The EM image should look like the testing EM.png." This description is too unspecific for novice users. Important would be here to emphasize that this is in context to the application of the pre-trained model on new data. 

**Retraining:**

 Great that the conda .yml is provided for easy installation of the dependencies. As a warning, I experienced that the creation of the conda environment took a long time on my machine. But could be specific to my setup. The possibilities of my review here are also limited since I do not have the possibility to generate my own training data from scratch and I can only test the provided data. Within these constraints, I was able to test the notebooks for their correct operation.

**Article:**

 The article is overall well written and explains the context of the work. The authors also test and discuss the limitations of the approach. Further, providing avenues to overcome problems and limitations. 

 As a side note, not relevant to the review, in terms of the registration field in the biology focused sciences, I am always a bit surprised that software solutions such as elastix (https://elastix.lumc.nl/) are not more widely used and discussed. I would have expected that the solutions of multimodal image registration developed in the medical field would be at least somewhat relevant to CLEM. The article seems to also discuss another established registration tool (eC-CLEM) so there is sufficient other context for this publication.

 The methods section of the article is unfortunately a bit sparse. The actual network architecture is only mentioned in the results. No further detail is provided. It is fine if the default values of the out-of-the-box network are used. However, this should be clearly stated in the methods. I would also wish a better description of how the training has been performed. Including how long the training took and on which hardware this was performed.

 I really enjoyed that the authors provide all necessary code and example images on their GitHub repo. The only tiny thing missing is to clearly state their preferred way of users getting in touch for support. I would suggest clearly stating this in the software availability section. 

 I endorse the article under the following conditions:

- A more extensive methods section. Describing clearer what the architecture was and any important or changed hyperparameters. Also, the hardware and the training time should be specified. 
- Improve documentation from the perspective of a novice user. 
- State how users should get in touch for support. 

Are the conclusions about the tool and its performance adequately supported by the findings presented in the article?

Yes

Is the rationale for developing the new software tool clearly explained?

Yes

Is the description of the software tool technically sound?

Yes

Are sufficient details of the code, methods and analysis (if applicable) provided to allow replication of the software development and its use by others?

Partly

Is sufficient information provided to allow interpretation of the expected output datasets and any results generated using the tool?

Yes

Reviewer Expertise:

Data science, bioimage analysis, cell biology.

I confirm that I have read this submission and believe that I have an appropriate level of expertise to confirm that it is of an acceptable scientific standard, however I have significant reservations, as outlined above.

# Reviewer report: Reviewer response for version 2

**DOI:** 10.5256/f1000research.136822.r146290

**Published:** 2022-08-30

**Authors:** Martin L. Jones

**Affiliations:** 1 Electron Microscopy Science Technology Platform, The Francis Crick Institute, London, UK

No further comments, the authors have covered all of the comments very satisfactorily.

Are the conclusions about the tool and its performance adequately supported by the findings presented in the article?

Partly

Is the rationale for developing the new software tool clearly explained?

Yes

Is the description of the software tool technically sound?

Partly

Are sufficient details of the code, methods and analysis (if applicable) provided to allow replication of the software development and its use by others?

Partly

Is sufficient information provided to allow interpretation of the expected output datasets and any results generated using the tool?

Yes

Reviewer Expertise:

Image Analysis, CLEM, Machine Learning, Electron microscopy

I confirm that I have read this submission and believe that I have an appropriate level of expertise to confirm that it is of an acceptable scientific standard.

# Reviewer report: Reviewer response for version 1

**DOI:** 10.5256/f1000research.30002.r77401

**Published:** 2021-02-08

**Authors:** Reinhard Rachel

**Affiliations:** 1 Centre for Electron Microscopy, Faculty of Biology and Preclinical Medicine, University of Regensburg, Regensburg, Germany

The manuscript by Seifert *et al. *(Univ. Würzburg, Germany) provides a convenient software tool (a macro name DeepCLEM) for linking fluorescent light microscopy images (here: structured illumination microscopy) to the information seen in electron micrographs (in this particular case: FE-SEM), obtained from serial sections on slides. 

 In a first glance, this short paper describes the development and application of a flexible and easy to handle software tool to a program which is open to many microscopy researchers, ImageJ / Fiji, in form of a macro, which can easily be added to any Fiji installation. Thus, it appears that this tool can be used by many groups aiming to analyze biological samples using correleative light and electron microscopy (CLEM). 

 A few questions arise when reading the manuscript - which do not modify the overall positive impression of this study. 

1. The group uses Hoechst 33342 for staining and the biological objects are visualized by SIM - would it be sufficient to visualize the objects by 'standard' wide-field fluorescent microscopy, which is present in almost all biological laboratories, while SIM requires access to instruments which are not available everywhere?
2. What kind of knowledge is necessary to perform the 'prediction of fluorescent signals from EM images', i.e. generating the "virtual" fluorescent images? Does this macro work as provided, i.e. 'fully automatically'? Is any user intervention needed? - the authors trained their macro on their datasets. Is it likely that this macro "as trained in the author's lab" also works on images from other labs? or is it necessary to go through a training phase? if so: workflow? 
3. Histogram equalization of EM images - was this done with routines implemented in Fiji?
4. Can the authors at least provide one set of parallel results obtained using DeepCLEM vs. ec-CLEM? or at least comment on this?

With comments on my points listed above, and according improvements in the manuscript, this can be indexed - thus, approved with reservations.

Are the conclusions about the tool and its performance adequately supported by the findings presented in the article?

Partly

Is the rationale for developing the new software tool clearly explained?

No

Is the description of the software tool technically sound?

Partly

Are sufficient details of the code, methods and analysis (if applicable) provided to allow replication of the software development and its use by others?

Partly

Is sufficient information provided to allow interpretation of the expected output datasets and any results generated using the tool?

Yes

Reviewer Expertise:

TEM, STEM, tomography, CLEM; microbiology, cell biology

I confirm that I have read this submission and believe that I have an appropriate level of expertise to confirm that it is of an acceptable scientific standard, however I have significant reservations, as outlined above.

# Response

**Published:** 2022-07-28

**Authors:** Philip Kollmannsberger

**Affiliations:** Julius-Maximilians-University Wurzburg, Germany

*R: The group uses Hoechst 33342 for staining and the biological objects are visualized by SIM - would it be sufficient to visualize the objects by 'standard' wide-field fluorescent microscopy, which is present in almost all biological laboratories, while SIM requires access to instruments which are not available everywhere?*

 A: If the structures used for alignment (e.g., chromatin) can be resolved and the WF images can be manually aligned, it is also possible to obtain a prediction and do automated alignment, although the achievable quality of the registration is obviously limited by the resolution of the fluorescence channel. We now added this in the discussion of the scope and limitations of the tool.

*R: What kind of knowledge is necessary to perform the 'prediction of fluorescent signals from EM images', i.e. generating the "virtual" fluorescent images? Does this macro work as provided, i.e. 'fully automatically'? Is any user intervention needed?*

 A: Using a trained network for prediction and alignment can be done fully automatically in Fiji, only the paths of the images and network must be provided in the user dialog. For retraining, some knowledge (e.g., setting up a python environment) is necessary. We now added a more detailed description of the training workflow. Currently, the training process should probably be performed by some more experienced person while using the trained network does not require any special knowledge.

*R: The authors trained their macro on their datasets. Is it likely that this macro "as trained in the author's lab" also works on images from other labs? or is it necessary to go through a training phase? if so: workflow?*

 A: It may be possible to use the provided network trained on our data on images from other labs directly, however in most cases re-training is required. We now describe the required workflow in more detail and perform a quantitative comparison of registration quality where images from an experiment were either included in the training set or not.

*R: Histogram equalization of EM images - was this done with routines implemented in Fiji?*

 A: We used the standard histogram equalization implemented in Fiji. We added this detail to the method description.

*R: Can the authors at least provide one set of parallel results obtained using DeepCLEM vs. ec-CLEM? or at least comment on this?*

 A: We now comment on ec-CLEM and its “autofinder” functionality which is closest to our tool in terms of functionality. While ec-CLEM autofinder uses spot finding or centers of segmented regions as points of interest to perform registration on, we predict the image of the other modality and then use correlation-based alignment.

# Reviewer report: Reviewer response for version 1

**DOI:** 10.5256/f1000research.30002.r77801

**Published:** 2021-02-04

**Authors:** Martin L. Jones

**Affiliations:** 1 Electron Microscopy Science Technology Platform, The Francis Crick Institute, London, UK

The authors present an automated method, deployed as a Fiji plugin, for registering 2D CLEM data. This is demonstrated on a "super resolution Array Tomography" (srAT) dataset, building on their previous method development work, where the sample is sliced into 100nm sections prior to imaging in SIM and SEM. The DeepCLEM plugin generates synthetic fluorescence data from the EM images that are aligned to the real fluorescence signal to perform the registration.

 This type of effort is crucial in harnessing the huge potential of correlative multimodal imaging as cross-modal registration is a significant bottleneck in many cases. The method of imaging both LM and EM on the processed and cut sections is perhaps not as widespread as other methods, such as, e.g. whole cell confocal imaging followed by resin-embedding, followed by EM imaging. The method used in this paper avoids the familiar CLEM registration problems of non-linear warping induced by the EM sample preparation and mismatched axial resolutions. This constrains the registration task to a "similarity" type transform rather than affine or non-rigid as is required in some other CLEM registration tasks.

 The open source plugin is easy to install and runs as described on the test data provided.

 A few points that I think should be addressed:

- In the abstract and introduction, it is mentioned that "registration is often done by hand using a fluorescent chromatin stain" - this seems overly specific, in general CLEM alignment might make use of many different markers or features. Focusing on the specific stains is appropriate later when describing the specific use-case presented, but the authors might inadvertently deter potential users wanting to use different stains that could feasibly be trained for in the deep learning step.
- The abstract refers to a Fiji Macro, but the code appears to be a python/jython script that is to be installed as a plugin. Is there an additional *.ijm IJ1 macro available, or is this a typo?
- Although it is common to call this type of method "automated", there is a front-loaded training step for the deep learning element. It isn't clear whether the trained model from the author's data would be sufficiently generalised to other datasets, or whether an amount of training will commonly be required for each different dataset.
- I believe the preferred capitalisation is "Fiji" and not "FIJI"
- ec-CLEM is mentioned and the text implies that it is only used with fiducials, but it can also be used for aligning using the fluorescence and EM data directly via manual landmark placement on intrinsic structures and/or stains. ec-CLEM also has an "autofinder" functionality that seems like it should be mentioned as potentially the nearest functionality in an existing tool. It would be useful to see a comparison, or an explanation as to how DeepCLEM differs if a direct comparison isn't possible.
- For the manual registration section, it would be better practice to use a more reproducible method, such as ec-CLEM or Fiji landmark-based registration (as opposed to the stated method using inkscape) since these methods retain a record of the steps and transforms. Although, in the context of the paper this is a means-to-an-end for acquiring training data for demonstration purposes, it would be preferable if the advice to potential users of the plugin were to err on the side of reproducible methods for their own training.
- In "Implementation" it would be useful to state a bit more detail about the training, for example training/validation split and suitable data quantities for users to train their own model.
- If the goal is to enable non-computational researchers to perform the whole workflow, the GitHub README and Jupyter notebooks should have a bit more description, in particular with regards to training, a process that is quite alien with unfamiliar jargon for non-specialists.
- The text mentions "correlation based alignment in Fiji" - looking in the source code this is specifically the "Register Virtual Stack Slices" plugin, this should be mentioned in the text as it is a major computational step in the workflow. Also, the use of "similarity" as opposed to rigid, affine etc should be specified in the text since other CLEM practitioners may be more familiar with other alternatives.
- An important point that is missing is some kind of quantification of the quality of the registration compared to a gold-standard produced manually. Understanding this is likely to be very important for researchers who might be considering using this tool. If possible, to maximise usability, some sort of "confidence" metric would be very useful for the user, but I appreciate this is probably non-trivial!
- The authors propose extensions to their method for dealing with situations where nuclei are not present in the images. It would be useful if the authors could provide a brief focused description of the scope of the tool as it is currently configured, so that potential users can quickly assess whether it's suitable for their task or what they might need to do to extend it. For example, the GitHub repo suggests the image must have a minimum of 3 nucleoli - this should be mentioned in the body of the paper as well. 
- Related to the above, the title and abstract make no specific mention of the method being based on imaging both LM and EM in the cut sections as opposed to a perhaps more common workflow such as confocal on whole cells followed by EM data on sections or block face. To avoid potential confusion, this constraint should be made more clear in the abstract.
- The abstract and discussion mention adaptations to apply to other modalities and 3D stacks. Given the srAT-based application it is not obvious what these adaptations would be, so an elaboration on this point would be useful

Overall, the tool itself works as described on the data type provided, but I think there is a risk that users might be tempted to stretch beyond the current scope of the tool, so this scope should be made more clear in the text.

Are the conclusions about the tool and its performance adequately supported by the findings presented in the article?

Partly

Is the rationale for developing the new software tool clearly explained?

Yes

Is the description of the software tool technically sound?

Partly

Are sufficient details of the code, methods and analysis (if applicable) provided to allow replication of the software development and its use by others?

Partly

Is sufficient information provided to allow interpretation of the expected output datasets and any results generated using the tool?

Yes

Reviewer Expertise:

Image Analysis, CLEM, Machine Learning, Electron microscopy

I confirm that I have read this submission and believe that I have an appropriate level of expertise to confirm that it is of an acceptable scientific standard, however I have significant reservations, as outlined above.

# Response

**Published:** 2022-07-28

**Authors:** Philip Kollmannsberger

**Affiliations:** Julius-Maximilians-University Wurzburg, Germany

*R: In the abstract and introduction, it is mentioned that "registration is often done by hand using a fluorescent chromatin stain" - this seems overly specific, in general CLEM alignment might make use of many different markers or features. Focusing on the specific stains is appropriate later when describing the specific use-case presented, but the authors might inadvertently deter potential users wanting to use different stains that could feasibly be trained for in the deep learning step.*

 A: We removed the mention of a specific stain in the abstract to reflect the general applicability of the method.

R: The abstract refers to a Fiji Macro, but the code appears to be a python/jython script that is to be installed as a plugin. Is there an additional *.ijm IJ1 macro available, or is this a typo?

 A: The code is a Jython script with graphical user interface to be run within Fiji. From the user's perspective, it is indeed a plugin. We changed “macro” to “plugin”, as suggested.

*R: Although it is common to call this type of method "automated", there is a front-loaded training step for the deep learning element. It isn't clear whether the trained model from the author's data would be sufficiently generalised to other datasets, or whether an amount of training will commonly be required for each different dataset.*

 A: The trained network can be applied to different datasets if they have similar contrast, usually from the same microscope or sample type. Otherwise, (re-)training of the network is necessary. We now make this clearer in the manuscript and include a description of the training workflow.

*R: I believe the preferred capitalisation is "Fiji" and not "FIJI"*

 A: Thank you, we corrected this.

*R: ec-CLEM is mentioned and the text implies that it is only used with fiducials, but it can also be used for aligning using the fluorescence and EM data directly via manual landmark placement on intrinsic structures and/or stains. ec-CLEM also has an "autofinder" functionality that seems like it should be mentioned as potentially the nearest functionality in an existing tool. It would be useful to see a comparison, or an explanation as to how DeepCLEM differs if a direct comparison isn't possible.*

 A: We now refer to ec-CLEM autofinder which is similar in terms of functionality. It is based on finding corresponding features in both modalities, e.g., using wavelet-based spot detection or the centers of segmented regions, and then performing registration of these automatically detected landmarks. We did not perform a direct quantitative comparison. If suitable corresponding spots can be found (e.g. beads), the performance on the same images using the same registration algorithm should be comparable. In cases where spot finding or similar methods in ec-CLEM do not perform well because the images are too dissimilar or if there are no fiducial markers, then transferring the EM image to fluorescence using DeepCLEM could be used to generate input data for point-based registration in ec-CLEM.

*R: For the manual registration section, it would be better practice to use a more reproducible method, such as ec-CLEM or Fiji landmark-based registration (as opposed to the stated method using inkscape) since these methods retain a record of the steps and transforms. Although, in the context of the paper this is a means-to-an-end for acquiring training data for demonstration purposes, it would be preferable if the advice to potential users of the plugin were to err on the side of reproducible methods for their own training.*

 A: We agree that these methods are more reproducible and added an additional note to the paragraph on manual registration to encourage users to use such reproducible methods. Since we already had created the training data, we did, however, not repeat the alignment. For assessing the performance (see below), we instead artificially displaced the two channels - in this case, the steps and transforms are also known exactly.

*R: In "Implementation" it would be useful to state a bit more detail about the training, for example training/validation split and suitable data quantities for users to train their own model.*

 A: We now include more detail about the training workflow in the implementation section and compared the performance for different amounts of training images.

*R: If the goal is to enable non-computational researchers to perform the whole workflow, the GitHub README and Jupyter notebooks should have a bit more description, in particular with regards to training, a process that is quite alien with unfamiliar jargon for non-specialists.*

 A: We extended both the description of the training parameters as well as the documentation of the entire workflow, including training in the Jupyter notebook.

*R: The text mentions "correlation based alignment in Fiji" - looking in the source code this is specifically the "Register Virtual Stack Slices" plugin, this should be mentioned in the text as it is a major computational step in the workflow. Also, the use of "similarity" as opposed to rigid, affine etc should be specified in the text since other CLEM practitioners may be more familiar with other alternatives.*

 A: The text now explicitly mentions the use of the "Register Virtual Stack Slices" plugin and the "similarity" transform in our work, but in principle, our workflow should also work with other means of correlation-based registration.

*R: An important point that is missing is some kind of quantification of the quality of the registration compared to a gold-standard produced manually. Understanding this is likely to be very important for researchers who might be considering using this tool. If possible, to maximise usability, some sort of "confidence" metric would be very useful for the user, but I appreciate this is probably non-trivial!*

 A: We now performed quantification of the quality of the registration using the manually aligned ground truth data as a reference where the best solution is already known. We observed that in most cases, the registration either works and has a very small error or is completely off by several 100 nm, which should be easy to spot. If a few images from an experiment are manually aligned and included in the training set, the registration of the remaining images improves significantly (Table 1).

 Instead of providing a confidence metric, which would indeed be non-trivial, we recommend visual inspection using the overlay. Possible ways to implement a confidence metric would be to output a confidence level for the neural network prediction, and to check the quality of the alignment using a correlation metric between fluorescent and predicted image.

*R: The authors propose extensions to their method for dealing with situations where nuclei are not present in the images. It would be useful if the authors could provide a brief focused description of the scope of the tool as it is currently configured, so that potential users can quickly assess whether it's suitable for their task or what they might need to do to extend it. For example, the GitHub repo suggests the image must have a minimum of 3 nucleoli - this should be mentioned in the body of the paper as well.*

 A: We added more detail regarding the scope and limitations of this tool and its current requirements to the abstract, introduction, and discussion section, including the need for at least three heterochromatin patches if chromatin staining is used for registration.

*R: Related to the above, the title and abstract make no specific mention of the method being based on imaging both LM and EM in the cut sections as opposed to a perhaps more common workflow such as confocal on whole cells followed by EM data on sections or block face. To avoid potential confusion, this constraint should be made more clear in the abstract. The abstract and discussion mention adaptations to apply to other modalities and 3D stacks. Given the srAT-based application it is not obvious what these adaptations would be, so an elaboration on this point would be useful*

 A: The way it is currently configured, DeepCLEM can in principle be used to register any CLEM data where corresponding 2D slices are available in both modalities, whether they were acquired on the same cut sections (srAT) or prior and post sectioning. The examples we provide however are restricted to srAT. We now state this in the text.
